# Evolution of University Students’ AI Dependency and Creativity in the Technology Diffusion Process: A Four-Wave Repeated Cross-Sectional Study in China

**DOI:** 10.3390/bs16101949

**Published:** 2026-10-20

**Authors:** Jianfeng Yin, Yafei Jin, Yang Yang

**Affiliations:** School of Education Science, Qingdao University, Qingdao 266071, China

**Keywords:** technology diffusion, external regulation, identified regulation, intrinsic motivation, AI dependency, AI-assisted creativity

## Abstract

Generative artificial intelligence is rapidly reshaping higher education, yet potential risks accompany over-reliance on these tools. Grounded in Rogers’ diffusion-of-innovations theory, the present study employed a mixed-methods design. Four waves of independent cross-sectional quantitative data (T1–T4, N = 788) were complemented by semi-structured interviews with 20 undergraduate students. Key patterns emerged as follows: (i) Across later waves, the direct association between external regulation and intrinsic motivation attenuated, while the indirect association operating via identified regulation strengthened. The path from identified regulation to intrinsic motivation was the only relationship that demonstrated significant cross-pair differences while satisfying invariance requirements for all three wave-pair comparisons. Because scalar invariance could not be established, this pattern is interpreted solely as a descriptive group-level temporal trend and should not be read as evidence of intra-individual change. (ii) AI-assisted creativity showed the largest and most consistent downward shift: estimates in later waves were significantly lower relative to the T1 baseline, with effect sizes increasing progressively (Δ = −0.391 to −0.549; d = −0.61 to −0.95; all *p* < 0.001 following Holm correction). This pattern remained largely unchanged after adjusting for gender, year of study, and academic major, and held up in four predefined subsamples as well as under propensity-score weighting. (iii) The linkage between AI dependency and creativity was significantly positive only at T3 and became negative but non-significant at T4, pointing to a conditional, wave-specific association rather than a stable linear relationship. (iv) Observed wave-level differences for AI dependency and intrinsic motivation did not withstand correction for multiple comparisons. Sensitivity tests yielded only two significant coefficients for AI dependency and none for intrinsic motivation; accordingly, these fluctuations are not given substantive theoretical interpretation. Retrospective qualitative accounts provided supplementary process-oriented context: participants reported initial exposure to AI driven by prevailing social trends, sustained use based on perceived utility, subsequent disappointment in unreliable or misleading outputs, and eventual shifts toward more strategic, self-regulated engagement. Given their retrospective nature, interview narratives were not used to triangulate or validate the quantitative findings. Collectively, this work suggests that as AI-supported learning becomes normalized within a diffusion-driven context, group-level indicators of students’ subjective creative experiences tend to decline. Higher-education institutions may therefore prioritize protecting learners’ creative agency as an explicit educational goal.

## 1. Introduction

Since ChatGPT’s rise, student over-reliance on AI has sparked concerns about declining creativity and critical thinking (D. Zhang et al., 2025). However, appropriate AI-supported learning can enhance intellectual autonomy and digital literacy (Sako, 2024). Because human-AI collaborative creativity is highly hybrid and paradoxical, it requires a human-centered approach (Baer et al., 2024), demanding that students develop autonomous, responsible motives and act as primary agents in AI-assisted learning (Vieriu & Petrea, 2025).

However, as an emerging technology, AI exerts highly dynamic and uncertain impacts throughout its diffusion. Particularly in China, this process has been dramatically accelerated by the sudden emergence of Generative AI at the beginning of 2025 (G. Wang, 2025; Xinhuanet, 2025). Following the rise of ChatGPT, this milestone marks the formal entry of AI technology into the Technology Takeoff stage of Innovation Diffusion Theory among Chinese university students (Rogers, 2003; Lechman, 2015). Against this backdrop of rapid technological diffusion, there is a growing need to explore evolutionary trends in students’ motivation for AI use, which may shed light on the balance between AI dependency and AI-assisted creativity.

Recent research has yielded divergent perspectives regarding the implications of AI utilization. On the one hand, a substantial body of scholarship warns that AI dependency may weaken learning motivation, foster academic inertia, and impair problem-solving, critical-thinking, and creative-thinking abilities (Morales-García et al., 2024; S. Zhang et al., 2024; Gerlich, 2025). On the other hand, empirical evidence has uncovered substantial potential for AI to support student creativity. For instance, experimental investigations have found no conclusive evidence that ChatGPT exerts a uniformly negative effect on creative thinking; instead, effects are heterogeneous, with some dimensions declining and others improving (Toma & Yánez-Pérez, 2024). Scholars have argued that although AI may erode creative confidence, it can effectively enhance student motivation and engagement when students maintain positive or tolerant attitudes toward AI, employ it to stimulate learning interest, explore personal meaning, and master its functional capabilities (Artemova, 2024; Habib et al., 2024; Pitts et al., 2025; Syifauddin & Yuliansyah, 2023). These findings collectively suggest that students’ perceived AI dependency is not only closely associated with learning motivation but also contingent on the nature of motivation toward AI. Moderate reliance or proficient usage tends to co-occur with more self-determined motivational orientations, which is consistent with predictions derived from Self-Determination Theory.

According to Self-Determination Theory (SDT), motivation can be described along a continuum from external control to autonomous self regulation, covering both intrinsic and extrinsic orientations (Ryan & Deci, 2000a). Similarly, students’ motivation to use AI in academic contexts follows this continuum of motivational evolution: starting with external regulation, driven by external pressure and efficiency seeking; progressing to identified regulation, in which students endorse the instrumental value of AI for personal academic development; and culminating in intrinsic motivation, rooted in genuine interest in AI itself or in the learning goals it supports. Consequently, whether an adaptive autonomous pattern or a maladaptive dependent pattern emerges in AI-supported learning (Cespedes, 2025) likely hinges on the degree to which students’ AI-related motivation becomes internalized.

Prior research often conflates general learning motivation with AI-specific usage motivation, overlooking how AI-driven external motivation internalizes. Moreover, while studies largely focus on AI’s detrimental effects on motivation and creativity (S. Zhang et al., 2024; Pitts et al., 2025), they rarely investigate whether moderate AI dependency and enhanced learning capabilities (Favero et al., 2025) might facilitate the transition from external to intrinsic motivation, thereby positively influencing AI-assisted creativity.

In summary, this study adopts a four-wave independent cross-sectional design, with separate samples of university students recruited at each wave. Drawing on Technology Diffusion Theory, Self-Determination Theory, and creativity theory, it explores group-level evolutionary trends of external, identified, and intrinsic motivational orientations and examines their associations with AI-assisted creativity amid the rapid diffusion of AI such as DeepSeek. A key innovation is its timely repeated cross-sectional design: the first wave (124 valid responses) was completed on 7 January 2025. Departing from static cross-sectional limitations, three additional repeated cross-sectional surveys were conducted at critical time points, supplemented by semi-structured interviews for triangulation. Cross-temporal comparisons were performed using SEM, MICOM, and multi-group analysis (MGA). This framework tracks dynamic trajectories of AI usage behaviors amid sudden technological shifts and explores how more self-determined motivational orientations may support autonomous, responsible AI-supported learning while mitigating educational risks. Specifically, this study addresses two core questions: (1) What are the levels of different motivational types and their associations amid technological diffusion? (2) What role does AI-use motivation play in the link between AI dependency and AI-assisted creativity?

### 1.1. Theoretical Background and Hypotheses

According to Rogers’ (2003) Diffusion of Innovations Theory, technology adoption follows an S-shaped curve and a five-stage individual process (Mbatha, 2024), shaped by social networks and uncertainty (Lechman, 2015). While traditional technologies took decades to diffuse, generative AI like ChatGPT has compressed this cycle to approximately one year (Dale, 2024), driven by efficiency, usability, perceived utility, and task-driven demand (Comin & Mestieri, 2014; Mukoyama, 2004).

For university students, AI adoption is driven by peer influence, developing proficiency, and task demands, with predictors including AI competence, intrinsic motivation, and perceived ease of use and usefulness (Galindo-Domínguez et al., 2025). Integrating this with Ryan and Deci’s (2000a) continuum of motivational autonomy, which describes that motivation can shift toward more self endorsed forms over time, the extrinsic incentives of AI adoption and learning motivation may exert reciprocal influences, with technology mediating the interplay between intrinsic motivation and external factors.

### 1.2. The Relationship Between AI Dependency and AI-Assisted Creativity

Creativity involves generating novel, valuable ideas (fluency, flexibility, originality) (Torrance, 1966). In this study, it is operationalized as AI-assisted creativity, namely university students’ perceived AI-assisted creativity. It describes university students’ subjective perception that they have generated creative academic solutions through interaction with AI. Because this hybrid model’s creative contributions are indeterminate, AI’s impact on student creativity is contingent on appropriate, responsible use (Sako, 2024). Excessive AI dependency may yield AI-dominated rather than genuine individual creativity, and insufficient understanding of AI can lead to irresponsible use (Baer et al., 2024). Thus, preserving human creative agency while preventing AI substitution is critical (Liao & Zhang, 2023).

AI dependency generally describes a state of over-reliance on artificial intelligence. In the present study, it is operationalized as AI dependency, namely university students’ perceived AI dependency. It refers to a psychological state in which satisfaction derived from using AI to complete challenging tasks leads university students to subjectively experience over-reliance and excessive trust in AI (Zhai et al., 2024).

In the present study, both AI-assisted creativity and AI dependency are obtained from university students’ self-reports. While AI may augment creative efficiency (Carroll, 2025), such perceived over-reliance can potentially erode knowledge accumulation and deep cognitive processing (S. Zhang et al., 2024). To investigate how balanced AI dependency enables AI-assisted creativity while preserving human cognition, we propose:

**Hypothesis 1.**  *AI dependency exerts a significant negative effect on AI-assisted creativity.*

### 1.3. The Mediating Role of External Regulation and Identified Regulation in AI Use

Based on the motivational continuum of Self-Determination Theory (Ryan & Deci, 2000a), this study adopts external regulation, identified regulation, and intrinsic motivation to capture students’ progression from non-autonomous AI dependency toward autonomous agency. Identified regulation—the most internalized form of extrinsic motivation (Ünlü, 2015)—encompasses perceptions of AI’s instrumental value together with enhanced learning aspirations.

Empirical evidence links AI dependency to lower academic motivation, learning inertia, cognitive laziness, reduced intrinsic motivation, and increased procrastination and boredom (Chen et al., 2025; Wu et al., 2025; Rudolph et al., 2024). As autonomy diminishes, students pursue short-term instrumental goals via AI, leading to superficial learning and hindering creativity. Creativity theory primarily attributes human creativity to intrinsic motivation, while extrinsic motivation may undermine it (B. Li et al., 2025; Schmidhuber, 2010), although extrinsic motivation moderates the self-efficacy-creativity relationship (Prabhu et al., 2008). While computational creativity is mostly extrinsically driven (Simo et al., 2020), intrinsic motivation remains central to AI-assisted artistic creation (Moura, 2018).

Accordingly, identified regulation may play a critical role, balancing student autonomy with AI’s extrinsic nature via quasi-moderating or intermediate functions. Conversely, external regulation emphasizes efficiency and instrumental value, prompting students to outsource cognitive processing for short-term goals (J. Li, 2022). This study explores how students integrate individual with AI-generated creativity by leveraging identified regulation’s autonomy, avoiding external regulation’s instrumentalism, and balancing AI dependency with autonomous learning. We propose:

**Hypothesis 2.**  *External regulation in AI use positively mediates the relationship between AI dependency and AI-assisted creativity.*

**Hypothesis 3.**  *Identified regulation in AI use positively mediates the relationship between AI dependency and AI-assisted creativity.*

### 1.4. The Mediating Role of Intrinsic Motivation in AI Use

Based on Self-Determination Theory (Ryan & Deci, 2000b), the intrinsic motivation in AI use employed in this study refers to two distinct types of psychological satisfaction experienced by university students in AI-assisted learning contexts: expectations and interest in AI technology itself, and expectations and interest in completing learning tasks. Since university students use AI in academic tasks, their motivation encompasses a mixture of technological interest and learning interest.

Numerous empirical studies have shown that the inherent difficulty of learning tasks motivates students to use technology; in turn, technology use reduces task difficulty, and appropriate or low task difficulty enhances learning motivation (Liao & Zhang, 2023; Reinhard & Dickhäuser, 2009). Therefore, university students may develop an interest in learning itself because AI use reduces the difficulty of learning tasks, thereby forming learning motivation (Mohamed et al., 2025; Schei et al., 2024). In this regard, to reduce the difficulty of learning tasks, students are likely to develop a sense of dependency on AI, which further increases their learning motivation and motivation to apply technology, thereby enhancing creativity in human-AI collaboration. Based on the above arguments, we propose the following hypothesis in the context of university students using AI to complete academic tasks:

**Hypothesis 4.**  *Intrinsic motivation in AI use positively mediates the relationship between AI dependency and AI-assisted creativity.*

### 1.5. The Serial Mediating Role of External Regulation, Identified Regulation, and Intrinsic Motivation in AI Use

Self-Determination Theory posits motivation as a continuum from non-autonomous to autonomous (Ryan & Deci, 2000a). We construct an AI use motivational spectrum—external regulation, identified regulation, and intrinsic motivation—describing how students internalize instrumental motives (AI convenience/efficiency) into genuine interest. Identified regulation, as autonomous extrinsic motivation, exerts positive effects (Ryan & Deci, 2000b); AI application positively facilitates motivation, and perceived ease of use synergizes with intrinsic motivation to promote usage intention (Hmoud et al., 2024; Afzaal et al., 2025). Initial AI competence also predicts initial usage motivation (Galindo-Domínguez et al., 2025). While the use of artificial intelligence is closely associated with intrinsic motivation among individuals, external motivation can also play a role. During tool acceptance and habit formation, moderate AI dependency under task pressure may promote active use, enhancing AI-assisted creativity. As proficiency deepens, students recognize AI’s learning value, replacing external incentives with identified regulation. Conversely, external regulation may weaken intrinsic interest, replacing it with technological advantages (Shvartsman et al., 2017), risking externalized, utilitarian motivation that impairs long-term experience and creativity (Wu et al., 2025).

Several scholars have suggested that AI dependency also comprises distinct stages: tool acceptance, habit formation, and psychological dependency (Favero et al., 2025), which lends support to a stance of moderate tolerance toward it (Pitts et al., 2025). Like motivation, AI dependency likely involves a non-autonomous to autonomous process. Nevertheless, AI serves as a learning partner enhancing personalized learning and creativity (Carroll, 2025; Kovari, 2025). Notably, as students accumulate experience, they gradually internalize AI-related motivation.

Drawing on the dual-effect perspective of perceived AI dependency and the change trajectory from external to autonomous regulation, we propose:

**Hypothesis 5.**  *External regulation, identified regulation, and intrinsic motivation in AI use exert a serial positive mediating effect on the relationship between AI dependency and AI-assisted creativity.*

**Figure 1:**
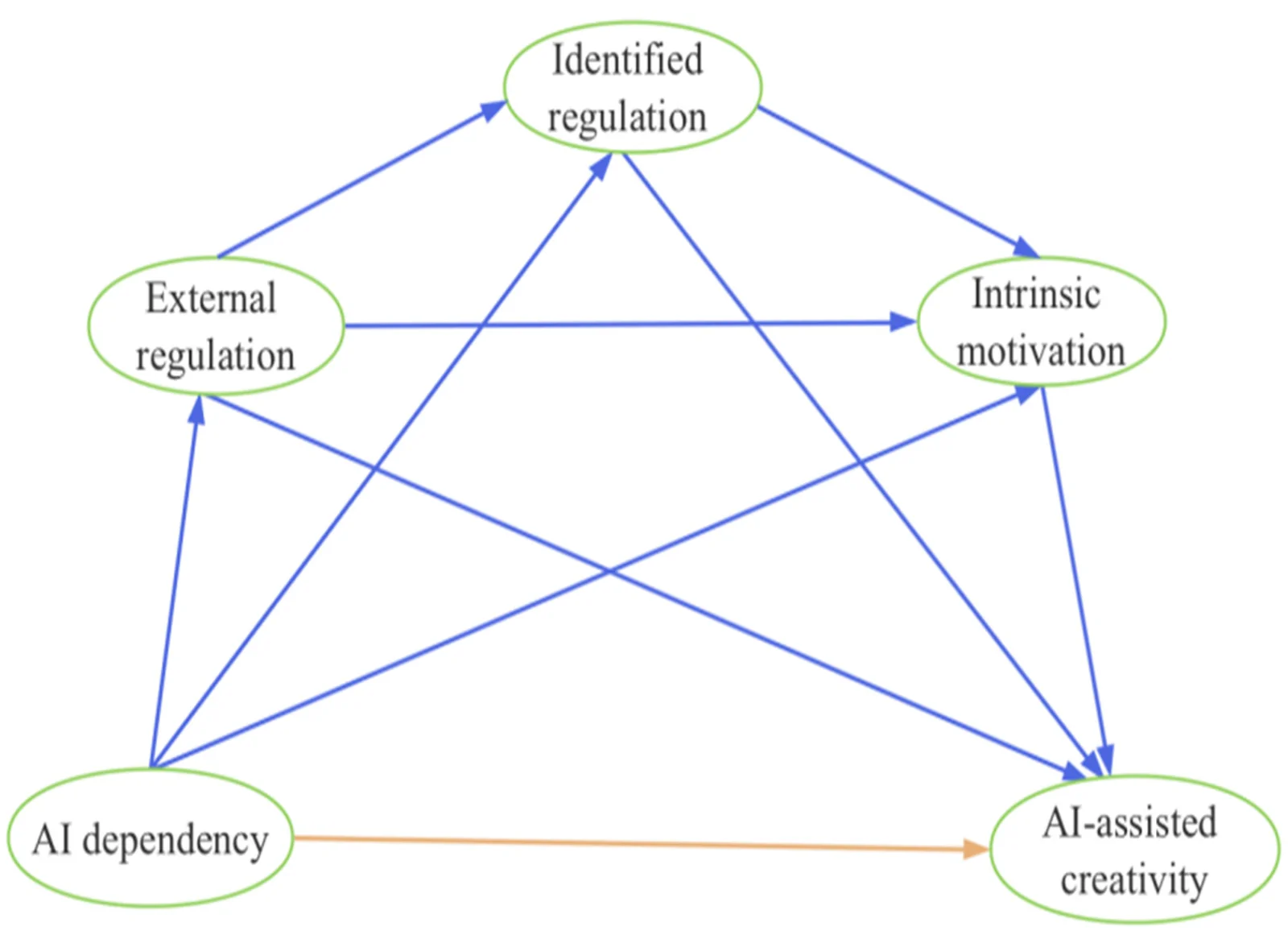
The hypothesized association model.

Guided by the above theoretical foundations, we develop the proposed research model as illustrated in Figure 1.

## 2. Methods

### 2.1. Methodology Flowchart

**Figure 2:**
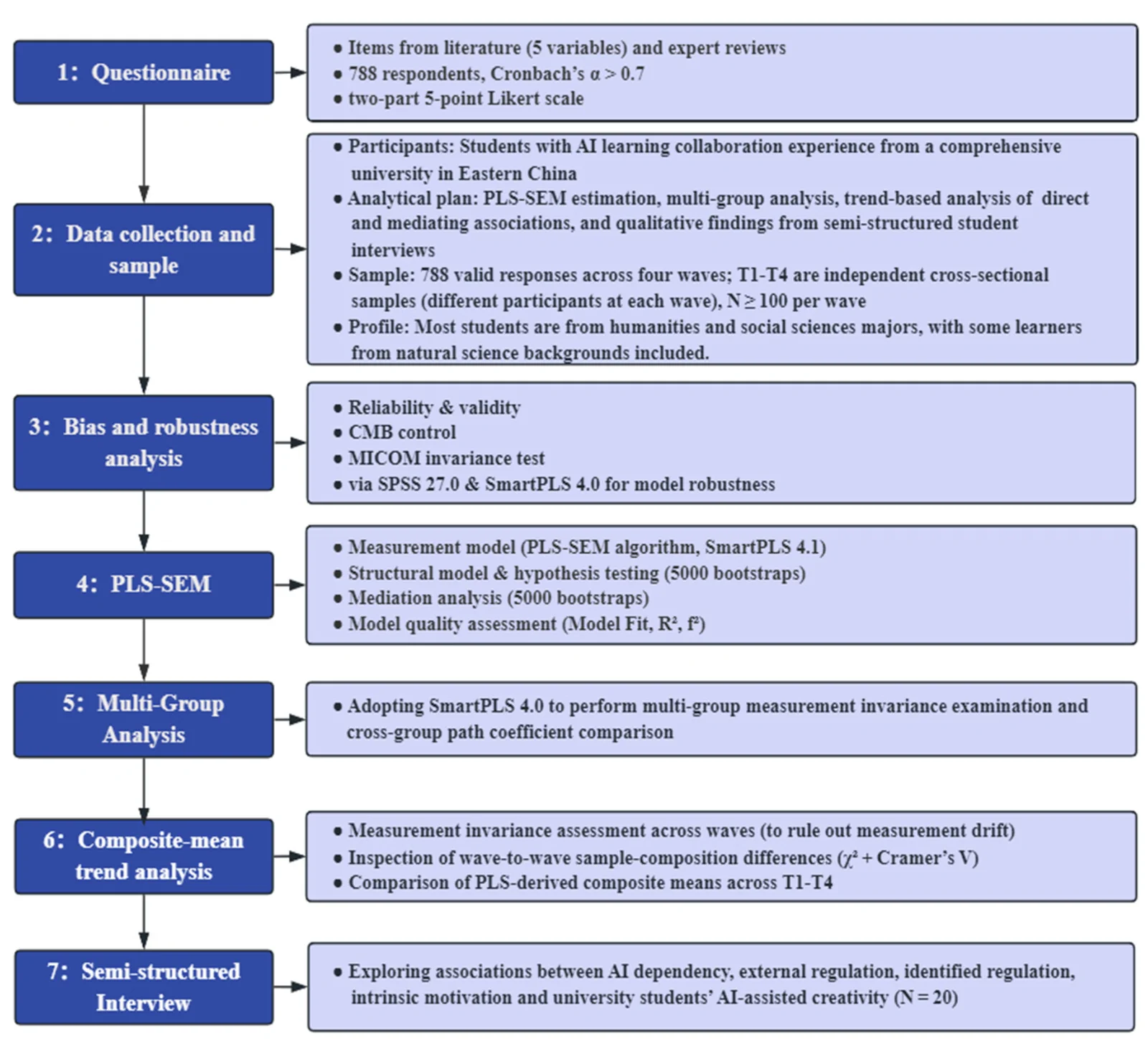
Research methodological flowchart. Note: T1–T4 represent four separate data-collection waves; each wave draws an independent cross-sectional sample with distinct participants, and this is not a panel design with repeated measurements of the same individuals. PLS-SEM = Partial Least Squares–Structural Equation Modelling; MICOM = Measurement Invariance of Composite Models.

Figure 2 illustrates the seven stages of the research on the long-term impacts of excessive AI dependency on university students’ learning motivation and creativity.

### 2.2. Measurement Design

This study used a 5-point Likert scale (1 = strongly disagree, 5 = strongly agree), adapting established scales to the AI-assisted learning context. Experts in psychology, educational technology, and AI evaluated face validity, and items were refined accordingly. Eligible participants were users of AI tools and provided informed consent. After a pilot test, the survey was distributed via the Wenjuanxing platform using snowball sampling, adhering to ethical guidelines to ensure data anonymity and confidentiality.

AI Dependency was measured using 5 items adapted from Morales-García et al. (2024), based on Andreassen et al. (2012) with localized revisions by Hu et al. (2023) and S. Zhang et al. (2024). While alternative methodologies exist—such as task performance, eye-tracking measures (integrated in a 2023 “Eyes are the Windows” framework), and statistical decision theory frameworks (Schoeffer et al., 2023; Guo et al., 2024)—this study employed subjective perception-based measures for contextual relevance.

AI-Assisted creativity was assessed using 10 items adapted from Torrance’s (1966) TTCT framework: Fluency (using AI to expand thinking), Flexibility (solving problems from multiple perspectives), and Originality (generating novel insights), tailored for human-AI collaboration.

Motivations were measured using 10 items adapted from Barkoukis et al. (2008) based on Self-Determination Theory (Deci & Ryan, 1985; Ryan & Deci, 2000a), contextualized for Chinese university students. It assessed three dimensions: External Regulation (driven by external rewards/norms), Identified Regulation (autonomous internal endorsement of academic utility), and Intrinsic Motivation (spontaneous engagement driven by inherent interest/enjoyment). Details are presented in Table 1.

### 2.3. Data Collection and Sample

This study used standardized questionnaires to examine how AI dependency influences AI-assisted creativity among undergraduates at a comprehensive university in Eastern China. Following the initial survey in January 2025, the rapid proliferation of AI in February significantly impacted higher education. To capture this event’s diffusion effect, a cross-sectional multi-group design with four independent, non-overlapping samples was adopted. A total of 788 valid questionnaires were retained (93.0% valid rate) across four waves:

- T1 (January 2025; *n* = 124): Baseline data.
- T2 (April 2025; *n* = 180): Initial AI adoption stage.
- T3 (October 2025; *n* = 258): Critical period for motivational evolution and creativity development.
- T4 (December 2025; *n* = 226): Stable state of long-term usage.

A total of 788 valid questionnaires were obtained across four independent non overlapping samples. Each sample exceeded the 100-participant minimum for multi-group PLS-SEM. Table 2 presents sample demographics and AI use characteristics with cross-wave distribution tests. Pearson χ^2^ tests showed significant compositional differences in gender, academic year and AI usage frequency, whereas academic major was balanced across waves. These variables were included as covariates in subsequent ANCOVA to reduce confounding, though residual bias cannot be fully ruled out.

### 2.4. Common Method Bias and Robustness

Harman’s single-factor test was first conducted as a preliminary check. The first unrotated factor accounted for 39.102% of the total variance, with four factors having eigenvalues greater than 1 (cumulative variance = 63.092%). However, this test can not definitively rule out common method bias.

We therefore adopted the full collinearity assessment approach recommended for PLS-SEM. Full collinearity VIF values for all latent variables ranged from 1.431 to 2.477, all below the conservative threshold of 3.3, suggesting that common method was not a major concern to the validity of the findings.

Notwithstanding these analytical efforts, we acknowledge that all core variables were collected from the same respondents through self-report questionnaires, which should be noted as an inherent limitation of this study.

### 2.5. Sensitivity Analyses

Given that the four waves relied on independent sampling and presented significant differences in background characteristics, six strategies were implemented to test the robustness of wave-related patterns against variations in sample composition. First, covariate adjustment: ANCOVAs were performed controlling for gender, year of study, and academic major using the full sample (*n* = 788). Strategies two through five comprised common-support subsample analyses, in which estimates were recalculated within more demographically homogeneous subgroups: (a) humanities-majoring students (*n* = 603); (b) female students (*n* = 640); (c) first-and second-year undergraduates (*n* = 448); and (d) female humanities-majoring students (*n* = 517). The humanities-only subsample is particularly informative, as no science-oriented students were included in Wave 1; this subsample eliminates perfect collinearity between academic major and wave membership. Sixth, propensity-score weighting: propensity scores were estimated with Wave 1 membership as the outcome and gender plus year of study as predictors. Non-Wave-1 observations were weighted by *p*/(1 − *p*) to align their demographic profile with that of Wave 1. Academic major could not be included in this weighting model due to the homogeneous major distribution in Wave 1. Holm correction was applied to wave-related contrasts across all analyses. Notably, the subsample of first-and second-year undergraduates contained only 56 participants in T3, representing a highly selected subgroup whose results should be treated as suggestive only. In addition, standard errors for the propensity-score-weighted estimates were not robust-adjusted; significance levels derived from these weights should therefore be interpreted with caution.

### 2.6. Analytical Technology

To explore group-level change trends in university students’ AI usage motivation across four time points (T1–T4), this study employed a progressive mixed-methods analytical approach. First, PLS-SEM was conducted using SPSS 27.0 and SmartPLS 4.0 to model the four-wave integrated data and examine hypotheses. Second, multi-group analysis (MGA) and trend analyses of direct and indirect associations were performed to examine temporal heterogeneity in path coefficients. Third, with Wave 1 set as the reference group, multi-group confirmatory factor analysis was conducted in R 4.5.2 to enable evaluation of composite-mean differences and group-level temporal trends for each construct across waves. Finally, semi-structured interviews were thematically coded to extract core themes, examining qualitative evidence alongside the quantitative findings.

## 3. Study 1: Testing of the Combined Four-Group Measurement Model

### 3.1. Reliability and Validity Analysis

This study assessed the questionnaire’s psychometric properties (Table 3). All item factor loadings exceeded the 0.6 acceptable threshold (most > 0.7), indicating adequate representation of latent variables. Reliability and convergent validity were supported, as Cronbach’s α (0.776–0.914), composite reliability (0.856–0.929), and AVE (0.568–0.757) all met recommended criteria. Discriminant validity was supported with all HTMT values below 0.85 (Table 4), and multicollinearity was not a concern with VIF values ranging from 1.389 to 2.630 (<3). Overall, the measurement model showed sound psychometric properties.

### 3.2. Correlation Analysis

Pearson correlation analysis showed significant positive correlations among all five variables (*p* < 0.01, Table 5). AI dependency was positively associated with AI-assisted creativity (r = 0.323). External regulation showed the strongest correlation with AI dependency (r = 0.534). At the motivational level, both identified regulation (r = 0.699) and intrinsic motivation (r = 0.652) were strongly correlated with creativity.

### 3.3. Model Fit Assessment

The structural model showed acceptable fit: SRMR = 0.062 (<0.08), NFI = 0.832. The coefficient of determination (R^2^) for AI-assisted creativity was 0.593. For the mediating variables, R^2^ values were 0.284 (external regulation), 0.238 (identified regulation), and 0.411 (intrinsic motivation), suggesting adequate explanatory power.

### 3.4. Path Analysis

**Figure 3:**
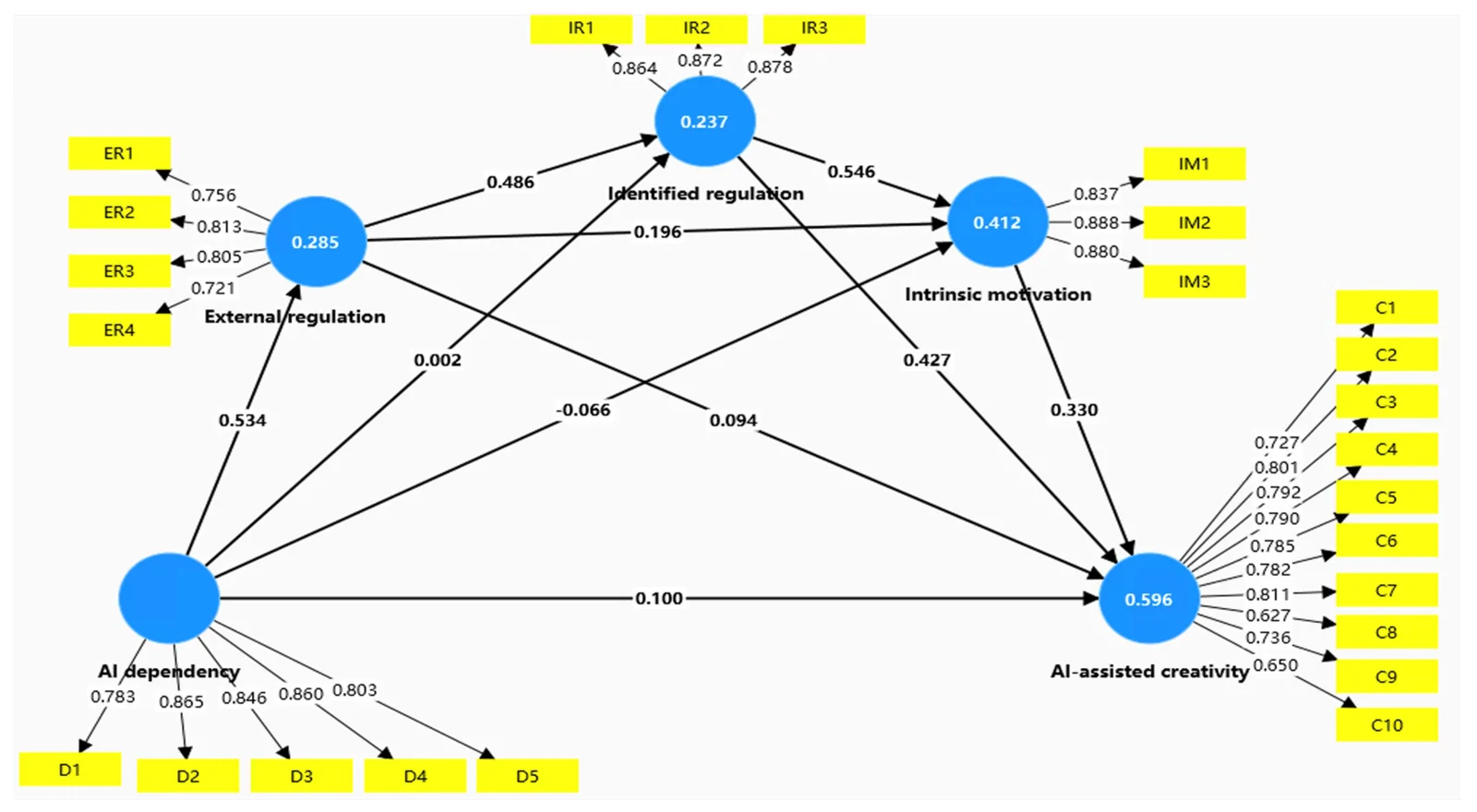
The tested association model with standardized parameter estimates.

As shown in Table 6 and Figure 3, the hypothesized direct association between AI dependency and AI-assisted creativity (H1) was not supported. Among the proposed indirect associations, only the two-step mediating association via external regulation (H2) and the full sequential multiple-mediation association running through external regulation, identified regulation, and intrinsic motivation in turn (H5) received empirical support. The mediating associations operating through identified regulation (H3) and intrinsic motivation (H4) were non-significant and could not be confirmed. Collectively, these observations suggest that AI dependency bears no direct association with AI-assisted creativity; its detectable association with AI-assisted creativity emerges only through this ordered sequence of motivational constructs.

Direct association tests showed a chain of motivational regulation associations (external regulation → identified regulation → intrinsic motivation), with all three positively associated with creativity. Indirect association analyses showed significant serial indirect associations: AI dependency → external regulation → identified regulation → intrinsic motivation → creativity. The two-step indirect association through external and identified regulation was most prominent (β = 0.111, 95% CI [0.036, 0.166]), followed by the indirect association through external and intrinsic motivation (β = 0.035, 95% CI [0.013, 0.089]) and the full three-step indirect association (β = 0.047, 95% CI [0.029, 0.070]). Notably, indirect associations not involving external regulation were insignificant, with their 95% CIs containing zero, suggesting external regulation as an initial intervening variable.

### 3.5. Sensitivity-Check Results

The wave-level difference in AI-assisted creativity persisted across all six sensitivity strategies (see Appendix A): the T4–T1 difference ranged from −0.478 (female-only subsample) to −0.650 (propensity-score weighting), with all *p* < 0.001 following Holm correction. Differences for T2 and T3 versus T1 were also significant in all six analyses (T2: −0.307 to −0.377; T3: −0.340 to −0.737). Notably, effect magnitude in the most restrictive “humanities × female” subsample (−0.499) was largely comparable to that in the full sample (−0.516), suggesting that this gap is unlikely to be accounted for by compositional differences in gender, year of study, or academic major.

Wave-level differences for identified regulation (T4) and external regulation (T3) also remained significant under all six analytical specifications (former: p_holm = 0.001–0.021, latter: p_holm = 0.001–0.013).

By contrast, the wave-level difference in AI dependency showed limited robustness: the T4 difference ranged from −0.181 to −0.288 across the six checks, and only two reached significance after Holm correction (p_holm = 0.039 and 0.005), with the remaining four falling between 0.064 and 0.40. Accordingly, we do not offer a substantive interpretation of cross-wave mean shifts in AI dependency.

## 4. Study 2: Multi-Group Analysis and Trend Analysis of Direct and Indirect Effects Across Four Time Points

This study employed the MICOM procedure to examine measurement invariance across the four time points (T1, T2, T3, T4). As shown in Appendix B, MICOM was conducted in three steps. Step 1 (configural invariance) was supported for all five constructs. Step 2 (compositional invariance) was supported for AI dependency, AI-assisted creativity, identified regulation, and intrinsic motivation across all six pairwise comparisons (all *p* > 0.05). External regulation, however, did not meet compositional invariance in T1–T3, T1–T4, T2–T3, and T3–T4 (all *p* < 0.05), and was supported only in T1–T2 and T2–T4. Step 3 (equality of means and variances) Among the time-point pairs that satisfied Step 2, the permutation *p*-values for composite means or variances were below 0.05 for most constructs, suggesting that only partial measurement invariance was achieved.

To examine whether path coefficients differ significantly across time points, this study conducted Bootstrap-based Multi-Group Analysis (MGA). Under the condition of partial measurement invariance, we extracted between-group differences in path coefficients with significant two-tailed *p*-values. The results showed substantial heterogeneity in structural path strengths across groups (Table 7). Combined with the trend analysis of direct and indirect associations across the four time points, the following findings were observed:

To control Type I error inflation due to multiple comparisons, we applied the Holm correction to pairwise comparisons of the proportion of effects across waves. Because the sum of the direct and indirect proportions is always 100%, they are complementary and yield equivalent tests (the two sets of *p*-values for the same pair in Table 8 are identical). Consequently, only six wave-pair combinations, rather than 12 independent tests, were included for correction. The results showed that only the T4-versus-T1 comparison remained significant after correction: the direct proportion decreased by 90.3% (95% CI [−166.4%, −37.9%], p_holm = 0.012), and the indirect proportion correspondingly increased by 90.3% (95% CI [37.9%, 166.4%], p_holm = 0.012). The remaining five pairs (T2 vs. T1, T3 vs. T1, T3 vs. T2, T4 vs. T2, T4 vs. T3) were non-significant after correction. Among them, T3 vs. T1 (*p* = 0.055) and T4 vs. T3 (*p* = 0.054) were marginally significant before correction, suggesting that differences between waves with shorter intervals are sensitive to sampling variation. Given that only one out of six pairs survived the correction and each wave comprised independent samples, cross-wave coefficient differences are reported below as descriptive trends across separate groups, and are not interpreted as evidence of change within the same group over time.

To characterize the relative contributions of direct and indirect effects together with their sampling uncertainty, we ran 10,000 bootstrap resamples for each wave separately and constructed confidence intervals for effect proportions using the percentile-based method (Table 9). In the T1 sample, the total effect of external regulation on intrinsic motivation was dominated by the direct path (direct proportion = 84.6%, 95% CI [51.3%, 112.8%]); the indirect proportion via identified regulation was non-significant (15.4%, 95% CI [−12.8%, 48.7%], containing zero). In the T4 sample, the point estimate of the direct proportion dropped to −5.7% (95% CI [−75.8%, 32.8%], containing zero), whereas the indirect proportion rose to 105.7% (95% CI [67.2%, 175.8%]). Proportions outside the range [0, 100%] occur when direct and indirect effects have opposite signs and represent a legitimate feature of this metric. Two caveats should be noted. First, since the confidence interval of the direct proportion still includes zero at T4, it is appropriate to describe this as “the point estimate is close to zero with limited estimation precision”, rather than stating that “the direct effect has disappeared”. Second, because external regulation failed the compositional invariance test across T1–T4 (Appendix A), these cross-wave comparisons serve only as descriptive references and do not provide sufficient evidence for cross-wave inferences.

At the level of path coefficients estimated independently for each wave (Table 10), the following group-level patterns can be observed. First, the direct path from external regulation to intrinsic motivation was significantly positive in the T1 sample (β = 0.465, *p* < 0.001) but non-significant in the T4 sample (β = 0.001, ns). The path from identified regulation to intrinsic motivation was non-significant in T1 (β = 0.139, ns), but became significantly positive across T2–T4 (β = 0.592, 0.612, 0.687, all *p* < 0.001). By contrast, the path from external regulation to identified regulation remained significant and relatively stable across all four waves (β = 0.584, 0.488, 0.483, 0.496, all *p* < 0.001). Second, among paths that passed the measurement invariance test, the link identified regulation → intrinsic motivation was the only one showing significant differences across waves for all three paired comparisons (T1–T2: Δ = −0.454, *p* < 0.01; T1–T3: Δ = −0.473, *p* < 0.001; T1–T4: Δ = −0.549, *p* < 0.001), constituting the most robust piece of cross-wave evidence in this study. Third, when calculating the proportion of the direct effect based on point estimates per wave, the values for the four waves are 85.2%, 36.2%, 46.1%, and 0.3%, showing a non-monotonic pattern—with an approximate 10-percentage-point rebound from T2 to T3. Therefore, the difference from T1 to T4 should not be summarized as a unidirectional “decline”.

**Figure 4:**
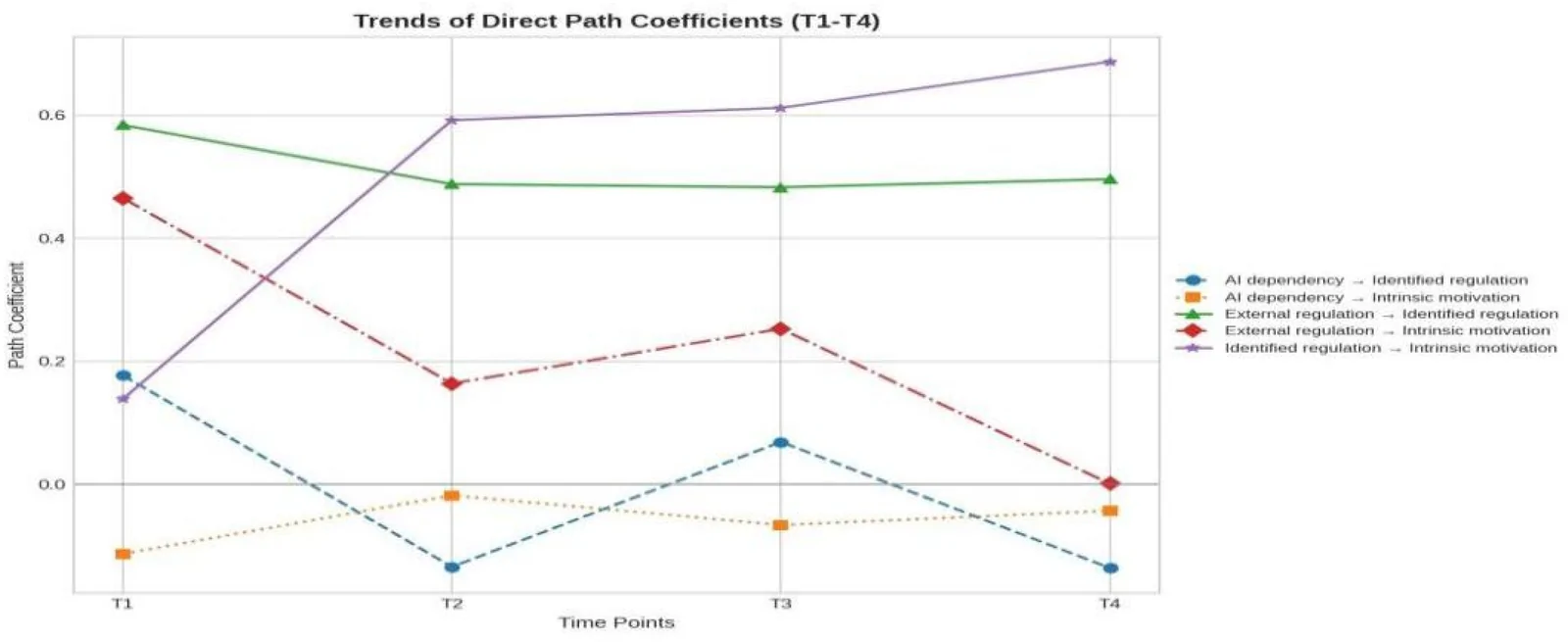
Trends of direct path coefficients concerning motivational orientations (T1–T4).

**Figure 5:**
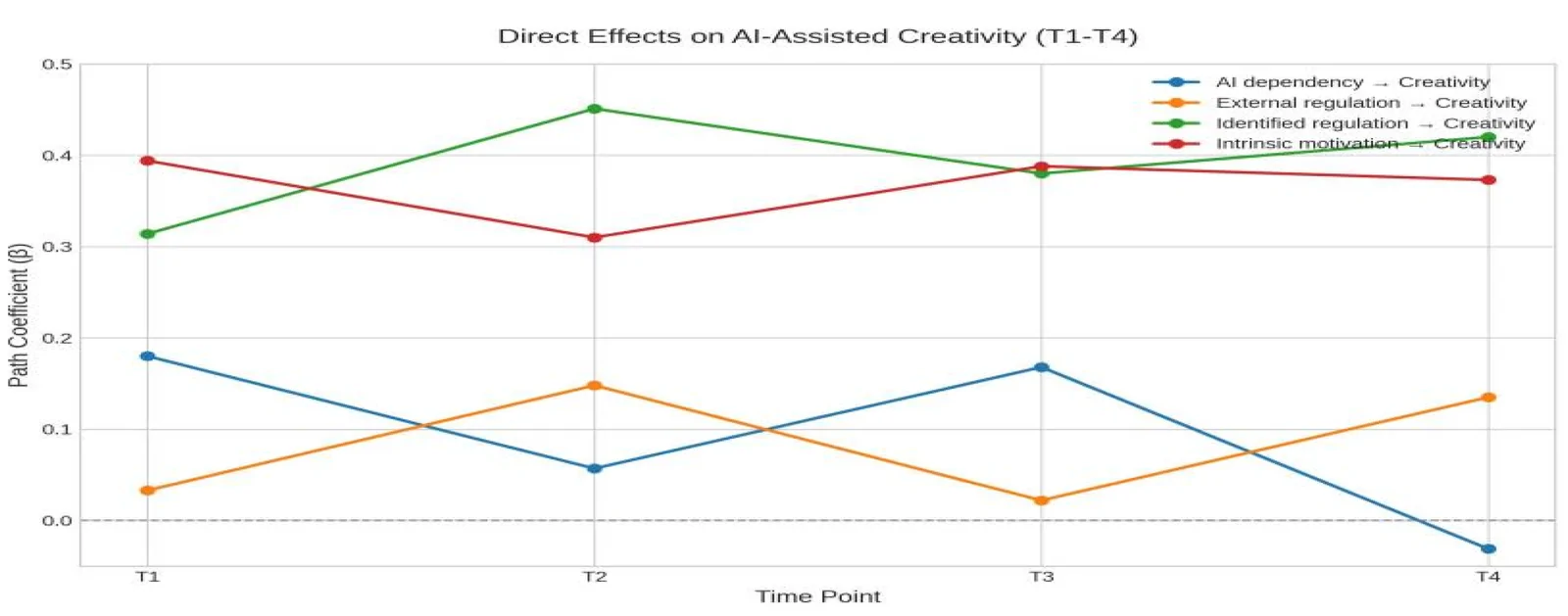
Direct effects on AI-assisted creativity (T1–T4).

**Figure 6:**
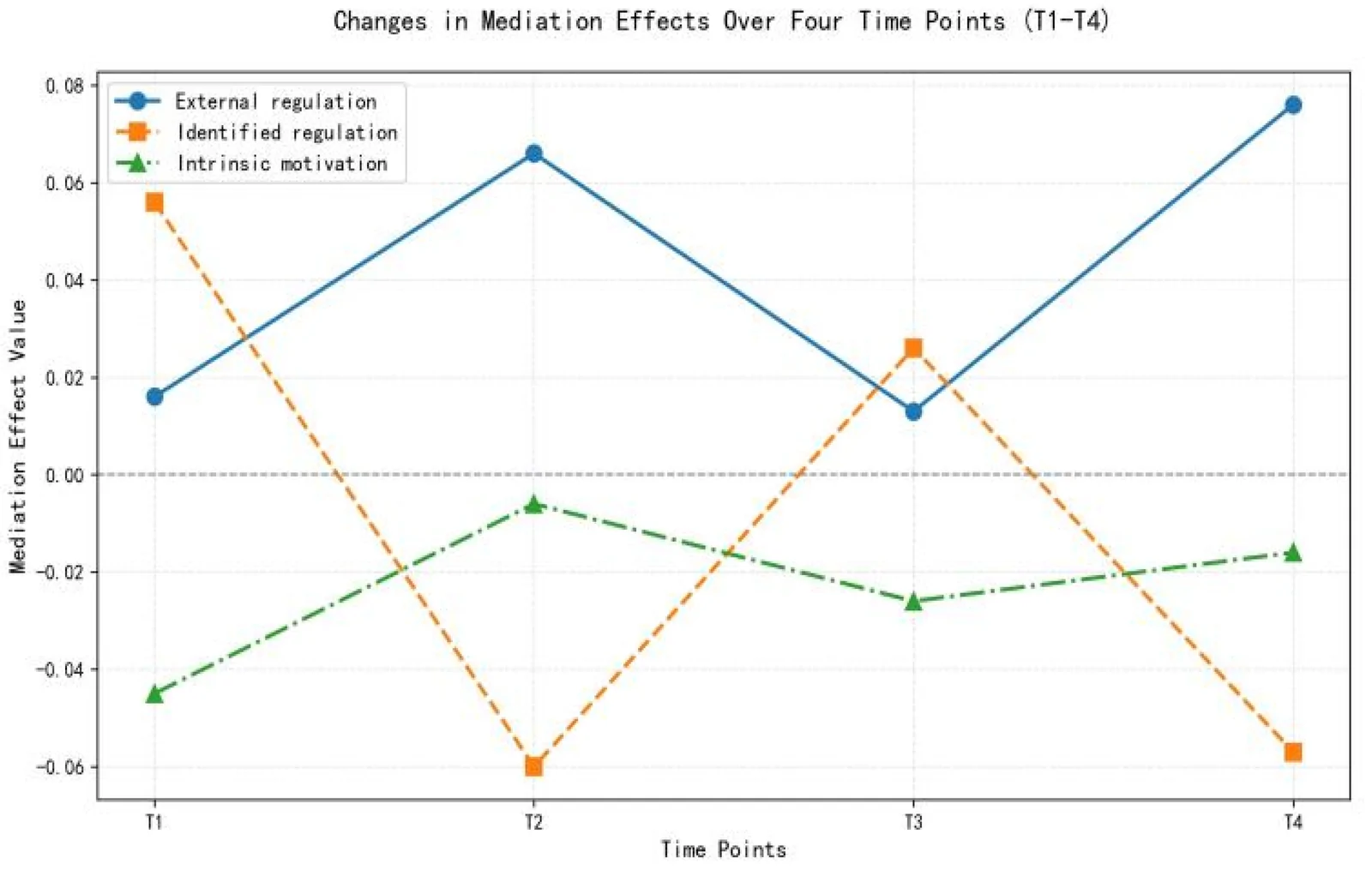
Changes in mediation over four time points (T1–T4).

Taken together, later-wave samples show a simultaneous “weakening of the direct association from external regulation to intrinsic motivation” and a “strengthening of the association from identified regulation to intrinsic motivation” (Figure 4, Figure 5 and Figure 6). This pattern aligns with the expected direction of motivational regulation shifting from externally controlled toward more autonomous forms proposed by Self-Determination Theory. Nevertheless, given independent sampling across waves, this study cannot track motivational changes within the same students, and the above results only indicate cross-group differences in association patterns at different time points.

Associations between different motivational types and perceived AI-assisted creativity exhibit heterogeneity (Table 10 and Table 11). Across the four wave-specific samples, identified regulation (β = 0.314, 0.451, 0.380, 0.420) and intrinsic motivation (β = 0.394, 0.310, 0.388, 0.373) consistently showed significant positive associations with relatively concentrated coefficient ranges. By contrast, the association of external regulation was unstable (β = 0.033 ns, 0.148, 0.022 ns, 0.135), reaching significance only in some waves. Mediation effects also displayed cross-wave differences: the simple mediation via external regulation was significant only in the T4 sample (0.016 ns → 0.066 ns → 0.013 ns → 0.076); the simple mediation via identified regulation and intrinsic motivation was non-significant in all four waves. In comparison, the three-path chained mediation via “external regulation → identified regulation → intrinsic motivation” became significant and showed increasing magnitudes in later-wave samples (0.015 ns → 0.040 → 0.067 → 0.071 **).

The direct association between perceived AI dependency and perceived AI-assisted creativity showed a fluctuating pattern across the four wave-specific samples (β = 0.180 ns, 0.057 ns, 0.168, −0.031 ns): it was significantly positive only in T3, the point estimate was negative but non-significant in T4, and the group-level difference between T3 and T4 was significant (Δ = 0.200, *p* < 0.05). Because this significant difference emerged only in a single pair, and the coefficient itself was non-significant in the later wave, it is appropriate to describe this as “the positive association between perceived AI dependency and creativity weakens and the point estimate turns negative in later-wave samples”, rather than claiming that over-reliance causes diminishing creative returns. Similarly, the direct association of Perceived AI dependency with identified regulation alternated between positive and negative values (0.177 → −0.134 ns → 0.069 ns → −0.136 ns), reaching significance only in the T1 sample.

## 5. Study 3: Composite-Mean Trend Analysis

### 5.1. Measurement Invariance Test

With T1 set as the reference group, configural, metric, and scalar invariance were tested sequentially for the three paired-wave comparisons. Metric invariance held across all three pairs (ΔCFI = 0.0022, 0.0009, 0.0055; all ≤ 0.010), indicating that factor loadings were comparable across waves and providing a measurement foundation for path-coefficient comparisons in Study 2. However, scalar invariance was not supported for any of the three pairs (ΔCFI = 0.0184, 0.0148, 0.0245; all > 0.010), suggesting that item intercepts differed across waves. Since latent-mean comparison requires scalar invariance as a precondition, we do not draw inferences based on latent-mean differences across waves; instead, we compare composite means at the observed level.

### 5.2. Wave-Level Differences in Sample Composition

The four waves consisted of independent samples, with significant differences in background characteristics across waves: gender χ^2^(3) = 25.14, *p* < 0.001, Cramer’s V = 0.179; major χ^2^(3) = 80.37, *p* < 0.001, V = 0.319 (no science-track students in T1); year of study χ^2^(12) = 413.91, *p* < 0.001, V = 0.418 (69.0% of T4 were first-year undergraduates; 41.5% of T3 were third-year undergraduates, and 31.8% of T3 were postgraduates). Accordingly, we tested wave-related differences after adjusting for these variables via ANCOVA.

### 5.3. Composite-Mean Comparisons Across Waves

As shown in Table 12 and Table 13, observed-level composite means for all five variables were lower in the later-wave samples relative to T1. AI-assisted creativity showed the largest and most robust decline (T2/T3/T4 differences = −0.391, −0.446, −0.549, all *p* < 0.001 after Holm correction, d = −0.61 to −0.95, with an increasing-magnitude pattern). Identified regulation decreased significantly at T4 (−0.395, p_holm = 0.003). All three wave differences for external regulation were negative; only T3 reached significance after correction (p_holm = 0.001), while T2 and T4 were at marginal levels (p_holm = 0.059 and 0.054). AI dependency exhibited similar negative differences across waves (−0.267 to −0.232, d ≈ −0.30), yet none were significant following Holm correction. Intrinsic motivation yielded the smallest, non-significant differences at all three comparisons (p_holm ≥ 0.550).

After adjusting for gender, year of study, and major, the magnitudes of wave-associated differences were largely preserved (average attenuation = 10.3%; 13 out of 15 comparisons changed by less than 30%), suggesting that the observed patterns were not attributable to compositional differences across samples.

Taken together, later-wave samples reported significantly lower scores on AI-assisted creativity relative to the baseline, with differences growing in magnitude across successive waves. Changes in AI dependency and intrinsic motivation were comparatively limited. Three important caveats should be emphasized. First, because scalar invariance was not established, these comparisons rely on observed-level composite means and cannot be interpreted as shifts at the latent-variable level. Second, the four waves draw independent samples; the detected differences reflect associational patterns between time points across distinct samples, rather than temporal change within the same group. Third, we did not conduct formal difference-in-differences tests to compare the magnitude of declines across variables; the ordering of decline sizes remains purely descriptive.

## 6. Study 4: Qualitative Insights from Student Interviews

To complement the quantitative findings and explore students’ experiences and interpretations of AI use, semi-structured focus group interviews were conducted after the completion of the four-wave survey. Using purposive sampling, 20 participants were selected from the survey respondents who volunteered for follow-up interviews. The sample consisted of 12 liberal arts undergraduates (S1–S12) and 8 master’s students (four liberal arts and four science students; P1–P8). All participants had continuously used generative AI for academic activities throughout the T1–T4 period. Three focus groups were conducted, including one undergraduate group (12 students, 2 h) and two postgraduate groups (4 students per group, 40 min each). It should be noted that these interviews are participants’ retrospective accounts, serving only as qualitative supplementation and not constituting longitudinal causal evidence (Table 14).

All interviews were conducted in Chinese, audio-recorded, and transcribed verbatim. The translated quotations presented in this article were independently translated and cross-checked by the authors to check consistency with the original meanings. Two researchers independently coded the transcripts, including initial open coding, theme development, and refinement through repeated comparison with the original data. Thematic analysis showed patterns the patterns of motivational change trends and their correspondence with the technology maturity curve. Beyond the core themes consistent with the quantitative results, three supplementary insights were identified from comparing undergraduate and graduate groups:

Both groups exhibited a similar pathway: initial exposure triggered by external trends, enthusiasm arising from perceived utility, disillusionment after encountering misinformation, and eventual deliberate normalization. Convenience-oriented habitual use rooted in extrinsic motivation persisted amid the trust crisis, alongside the development of strategies such as cross-verifying information and repositioning AI as an assistive tool. It should be noted that the continuation of such usage behavior does not equate to stable motivation levels -quantitative results show that the composite mean of external regulation also decreased in later-wave samples (significant at T3, p_holm = 0.001).

Undergraduates report intense emotional experiences, leaning toward entertainment and companionship, alongside pronounced anxiety over cognitive dependency. Conversely, master’s students display stronger task orientation and instrumental rationality, systematically adopting AI for academic support (e.g., literature reviews) and focusing strategic adjustments on methodological optimization and human oversight, consistent with motivational change trends and more balanced human-AI strategies. Because the undergraduate sample comprised exclusively humanities students, while the postgraduate sample included both humanities and science students, the observed differences may reflect disparities in both educational level and disciplinary training; the present study cannot disentangle these two sources.

Both groups grapple with balancing AI reliance for efficiency against skepticism regarding its reliability and cognitive risks. Students consciously employ mitigation strategies, suggesting a shared challenge in managing the trade-offs of AI integration in learning.

## 7. Discussion

Based on four-wave data and against the backdrop of the rise of generative AI, this study integrates Rogers’ (2003) diffusion model and the theoretical framework proposed by Ryan and Deci (2017). Using a multi-wave repeated cross-sectional design, it examines motivational change trends in university students’ AI use and their dynamic associations with perceived AI-assisted creativity.

First, the results reveal a change trend from external regulation toward identified regulation. Based on independently sampled university students at each wave, the share of the direct path from external regulation to intrinsic motivation decreased from 85.2% to 0.3% (T1–T2), while its indirect effect via identified regulation surged to 99.7%. This confirms external motivation is short-lived in AI learning (J. Li, 2022), whereas intrinsic motivation remains sufficiently resilient (Afzaal et al., 2025; Artemova, 2024; Martín-Núñez et al., 2023), aligning with classic theories on diminishing external rewards (Eisenberger & Cameron, 1996).

Second, this study situates motivational change trends within the context of ChatGPT diffusion (Dale, 2024). External factors exhibited a fluctuating pattern, whereas internal factors showed an opposite trend. Post-hoc retrospective narratives from interview participants suggest that university students’ initial strong enthusiasm and curiosity toward generative AI gradually became more rational, accompanied by readjustments in their AI-use behaviors. This aligns with the view that human agency should take the lead in human-AI collaboration (Baer et al., 2024).

Third, this study extends beyond the conventional view that perceived AI dependency is negatively associated with creativity, revealing a weak positive predictive association based on observed-level composite means. Over-reliance may be linked to diminishing-return patterns (Pitts et al., 2025). Furthermore, when cognitive-oriented dependency exceeds instrumental-oriented dependency, it may show a negative association with creativity (Yang et al., 2025). Mediation trajectories differ: the association involving external regulation shows descriptive dynamic trends, whereas identified regulation displays an opposite descriptive dynamic trend. Qualitative data align with the tension between efficiency and deep understanding, which is consistent with the crowding-out effect (Frey & Oberholzer-Gee, 1997). Cognitive-offloading anxiety is associated with students’ defensive restriction of AI use.

### 7.1. Implications

This study adopts a repeated cross-sectional mixed methods design and proposes an integrative framework linking diffusion-of-technology theory and self-determination theory. Three main contributions are outlined. First, it offers new evidence for self-determination theory: among the paths for which measurement invariance held, the association from identified regulation to intrinsic motivation became significantly stronger in later-wave samples (significant across all three paired comparisons, Δ = −0.454 to −0.549). Meanwhile, the direct association from external regulation to intrinsic motivation shifted from significant (β = 0.465) to non-significant (β = 0.001, ns). This pattern aligns with the expectation that motivational regulation shifts from externally controlled orientations toward more autonomous orientations, corresponding to the “technology take-off” phase (Lechman, 2015) and the new framework for AI-supported learning (Y. Wang & Wind, 2022). It should be noted that values based on effect proportions (e.g., the direct proportion dropping to 0.3%) are constrained by measurement invariance for external regulation and serve only descriptive purposes. Second, path associations linked to extrinsic drivers were stronger in earlier waves and weakened in later waves, whereas the path associated with intrinsic drivers (identified regulation → intrinsic motivation) grew stronger. Interview narratives depicted high initial expectations at the onset of AI use, followed by disappointment after encountering inaccurate AI-generated information, and finally rational recalibration—providing a plausible account for these wave-related differences. It is important to emphasize that this congruent pattern is reflected in wave-based differences in path coefficients, not in rises or falls of observed-level composite means: the observed-level composite means of AI dependency differed little across the three later-wave samples and showed no peaks or troughs. Finally, this study refines the applicability of continuance-use theory (Bhattacherjee, 2001) within the generative-AI context. In later-wave samples, self-evaluated AI-assisted creativity decreased significantly, while changes in the levels of AI dependency and intrinsic motivation were small and non-significant. This suggests that “continuance of use” does not necessarily accompany improvements in subjective creative experience—sustained usage and declining experiential evaluations can co-occur. Qualitative data further indicate that students continued to use AI for convenience even after the trust crisis, yet engaged in self-regulation via strategies such as cross-verifying information and setting usage boundaries. This reveals that continuance of use may involve active boundary management rather than passive AI dependency.

At the practical level, universities can implement a phased instructional strategy. For novice users, institutions should lower the barriers to AI adoption and accommodate initial external motivation to foster an open exploratory mindset (Pitts et al., 2025), and further cultivate learning interest through diversified learning tasks (Tiwari et al., 2024). Given the essential role of identified regulation, case teaching and reflective teaching should be adopted to channel external motivation toward creativity conducive development and strengthen intrinsic motivation (Artemova, 2024). After students accumulate sufficient AI learning experience, universities should provide structured guidance and ethical norms (Scholl & Kiesler, 2024) and integrate AI into diverse learning activities to alleviate cognitive offloading (L. Zhang & Xu, 2025). When students fully master human AI collaborative learning skills, educators should focus on potential risks of cognitive AI dependency and learning burnout (Lan et al., 2026), shifting instructional emphasis to metacognitive competence and interactive ability to cultivate student autonomy (Kim et al., 2026). Furthermore, given that the decline in AI assisted creativity is the largest and most robust across all variables, universities prioritizing AI integrated teaching should focus on protecting students’ creative subjectivity rather than merely emphasizing usage frequency or dependency level. Educational practices including setting AI free learning sessions, requiring explicit disclosure of AI contribution ratios, and adopting process evaluation instead of outcome evaluation can prevent instrumental AI usage from crowding out independent and original thinking.

### 7.2. Limitations and Future Research

This study has several limitations that should be addressed in future research. First, the findings rely entirely on self-reported questionnaire data, which may be associated with subjective bias and common method variance. In particular, the subjective measurement of AI-assisted creativity may complicate accurate assessment of actual creative performance, because participants’ perceived creativity does not necessarily correspond to their real creative output. Future research should therefore incorporate objective creativity measures to complement subjective questionnaire evidence and strengthen analytical robustness. Second, this study uses four waves of independent cross-sectional data collected at different time points. This design is suitable for exploring and describing group-level temporal trends in students’ AI-use motivation, yet independent sampling across waves constitutes an inherent boundary that prevents us from examining within-individual dynamic change. Although some effect sizes are small (for example, the small f^2^ for the direct association between AI dependency and creativity), these findings may still carry practical implications. Statistical significance is not equivalent to substantive significance, and the observed patterns remain meaningful for AI-supported learning. Third, another limitation concerns reverse causality. For instance, students with higher creativity may be more inclined to use AI, rather than AI dependency shaping creativity. Given the repeated cross-sectional design with distinct samples in each wave, the direction of these associations cannot be rigorously identified. Accordingly, conclusions should be interpreted as group-level associational patterns, not causal effects. Fourth, this study was conducted against the backdrop of the sudden popularity of generative AI, a specific external technological event. The uniqueness of this context may limit the general replicability of the present findings. Fifth, there were substantial differences in gender, discipline, and grade composition across waves. Although these variables were controlled via ANCOVA, residual confounding cannot be fully ruled out. There are also constraints related to measurement invariance and mean comparisons; wave-level differences in AI dependency are sensitive to sample composition. Future studies may further test the stability of these results using conventional AI tools characterized by gradual development. Finally, all participants were recruited from a single university, restricting sample diversity. The relatively homogeneous participant pool may limit the generalizability of conclusions to broader student populations. Given the female-skewed gender distribution in this sample, future research could expand sampling to include participants from different types of higher-education institutions.

## Figures and Tables

**Table 1:** Measurement.

Questions		References
AI dependency	I feel less confident when completing academic tasks without the assistance of AI tools. I find it increasingly difficult to finish assignments independently and tend to rely on AI for answers. When encountering learning difficulties, my first reaction is to turn to AI tools rather than solve problems on my own. After using AI tools, I engage in less in-depth reading and thinking. I find that my ability to solve complex problems has declined without the help of AI.	Andreassen et al. (2012), Hu et al. (2023), S. Zhang et al. (2024), Morales-García et al. (2024)
AI-assisted creativity	Even when time is limited, I can quickly generate multiple answers using generative AI. I frequently use generative AI to provide a wide range of ideas and solutions. I find that using generative AI helps me generate more thoughts when thinking about problems. I can adjust my inputs to generative AI to obtain different types and styles of answers. When using generative AI, I can explore solutions to problems from multiple perspectives. When generative AI outputs do not meet my expectations, I can flexibly adjust my strategy to find appropriate answers. I can often guide generative AI to help me develop new approaches to problem-solving. My answers are often considered unique and innovative by classmates, thanks to the assistance of generative AI. The answers I generate using generative AI often contain novel viewpoints and insights. I can use generative AI to explore solutions that have not been considered by others.	Torrance (1966)
External regulation	I use AI learning tools because I do not want to fall behind classmates who use them. I use AI learning tools because I want to achieve good grades in courses and exams. I use AI learning tools because the school encourages us to use them. I use AI learning tools because I want to earn more credits or rewards through them.	Deci and Ryan (1985), Ryan and Deci (2000a), Barkoukis et al. (2008)
Identified regulation	I believe using AI learning tools is important for my academic growth and future career development. I use AI learning tools because I feel I should utilize all available resources for learning. I use AI learning tools because I recognize their value in improving my learning efficiency.	
Intrinsic motivation	I use AI learning tools because I find the learning process itself enjoyable. I feel engaged when using AI learning tools and do not mind spending time on it. I find it enjoyable to explore new knowledge using AI learning tools.	

**Table 2:** Sample demographic and AI-use characteristics across four measurement time points, with cross-wave distribution tests.

Characteristics	Category	T1 (*n* = 124)		T2 (*n* = 180)		T3 (*n* = 258)		T4 (*n* = 226)		Cross-Wave Distribution Test χ^2^, *p*
		Count (*n*)	Percentage (%)	Count (*n*)	Percentage (%)	Count (*n*)	Percentage (%)	Count (*n*)	Percentage (%)	
Gender	Male	10	8.1%	35	19.4%	39	15.1%	64	28.3%	χ^2^ = 20.43, *p* < 0.001
	Female	114	91.9%	145	80.6%	219	84.9%	162	71.7%	
Academic year	Freshman	76	61.3%	47	26.1%	15	5.8%	156	69.0%	χ^2^ = 54.72, *p* < 0.001
	Sophomore	14	11.3%	52	28.9%	41	15.9%	47	20.8%	
	Junior	27	21.8%	72	40.0%	107	41.5%	3	1.3%	
	Senior	0	0.0%	0	0.0%	13	5.0%	16	7.1%	
	Graduate	7	5.6%	9	5.0%	82	31.8%	4	1.8%	
Academic major	Humanities and Social Sciences	107	86.3%	158	87.8%	194	75.2%	144	63.7%	χ^2^ = 10.17, *p* = 0.118
	Natural Sciences	17	13.7%	22	12.2%	64	24.8%	82	36.3%	
AI usage frequency	0–2 times/month	19	15.3%	21	11.6%	25	9.7%	21	9.3%	χ^2^ = 96.90, *p* < 0.001
	3–5 times/month	48	38.7%	113	62.8%	181	70.2%	168	74.3%	
	6–10 times/month	50	40.3%	33	18.3%	46	17.8%	32	14.2%	
	>10 times/month	7	5.6%	13	7.2%	6	2.3%	5	2.2%	

Note: T1–T4 are four independent cross-sectional samples with distinct participants.

**Table 3:** Reliability and convergent validity.

Construct	Items	Loadings >0.7	VIF <5	Cronbach’α >0.7	Composite Reliability >0.7	AVE >0.5
AI dependency	D1	0.783	1.719	0.888	0.918	0.691
	D2	0.865	2.580			
	D3	0.846	2.393			
	D4	0.860	2.630			
	D5	0.803	2.123			
AI-assisted creativity	C1	0.729	2.004	0.914	0.929	0.568
	C2	0.803	2.528			
	C3	0.794	2.447			
	C4	0.792	2.371			
	C5	0.780	2.193			
	C6	0.784	2.381			
	C7	0.813	2.569			
	C8	0.627	1.815			
	C9	0.739	2.076			
	C10	0.650	1.710			
External regulation	ER1	0.756	1.527	0.776	0.856	0.598
	ER2	0.805	1.572			
	ER3	0.721	1.389			
	ER4	0.813	1.667			
Identified regulation	IR1	0.864	1.898	0.839	0.903	0.757
	IR2	0.872	2.018			
	IR3	0.878	2.019			
Intrinsic motivation	IM1	0.837	1.667	0.836	0.901	0.752
	IM2	0.888	2.226			
	IM3	0.880	1.870			

**Table 4:** Assessment of discriminant validity using HTMT.

	AI Dependency	AI-Assisted Creativity	External Regulation	Identified Regulation	Intrinsic Motivation
AI dependency					
AI-assisted creativity	0.358				
External regulation	0.640	0.580			
Identified regulation	0.301	0.797	0.595		
Intrinsic motivation	0.208	0.745	0.526	0.733	

**Table 5:** Means, standard deviations, and correlations of the study constructs.

	M	SD	AI Dependency	AI-Assisted Creativity	External Regulation	Identified Regulation	Intrinsic Motivation
AI dependency	3.087	0.796	1				
AI-assisted creativity	3.597	0.637	0.323 **	1			
External regulation	3.271	0.702	0.534 **	0.491 **	1		
Identified regulation	3.786	0.712	0.260 **	0.699 **	0.478 **	1	
Intrinsic motivation	3.510	0.772	0.178 **	0.652 **	0.420 **	0.610 **	1

Note. ** *p* < 0.01, two-tailed.

**Table 6:** Path analysis results.

Path	β	SD	*p*	f^2^	95% Bootstrap CI	Support
H1: AI dependency→AI-assisted creativity	0.100 ***	0.033	0.002	0.018	[0.036, 0.166]	Not Support
AI dependency→External regulation	0.534 ***	0.031	0.000	0.399	[0.471, 0.594]	-
AI dependency→Identified regulation	0.002 ns	0.043	0.965	0.000	[−0.082, 0.086]	-
AI dependency→Intrinsic motivation	−0.066 ns	0.043	0.122	0.005	[−0.150, 0.019]	-
External regulation→AI-assisted creativity	0.094 *	0.037	0.012	0.012	[0.023, 0.165]	-
External regulation→Identified regulation	0.486 ***	0.045	0.000	0.221	[0.397, 0.575]	-
External regulation→Intrinsic motivation	0.196 ***	0.047	0.000	0.038	[0.100, 0.287]	-
Identified regulation→Intrinsic motivation	0.546 ***	0.038	0.000	0.386	[0.470, 0.619]	-
Identified regulation→AI-assisted creativity	0.427 ***	0.046	0.000	0.248	[0.333, 0.516]	-
Intrinsic motivation→AI-assisted creativity	0.330 ***	0.048	0.000	0.159	[0.240, 0.431]	-
H2: AI dependency→External regulation→AI-assisted creativity	0.050 *	0.020	0.012	-	[0.013, 0.089]	Support
H3: AI dependency→Identified regulation→AI-assisted creativity	0.001 ns	0.019	0.965	-	[−0.035, 0.037]	Not Support
H4: AI dependency→Intrinsic motivation→AI-assisted creativity	−0.022 ns	0.014	0.109	-	[−0.048, 0.007]	Not Support
H5: AI dependency→External regulation→Identified regulation →Intrinsic motivation→AI-assisted creativity	0.047 ***	0.010	0.000	-	[0.029, 0.070]	Support

Note: *** *p* < 0.001, * *p* < 0.05, ns = not significant (*p* ≥ 0.05).

**Table 7:** Difference and Two-tailed *p*-values for group comparisons of path coefficients.

Path	T1–T2	T2–T3	T3–T4	T2–T4	T1–T3	T1–T4
AI dependency→AI-assisted creativity			0.200 *			0.211 *ms*
AI dependency→External regulation						
AI dependency→Identified regulation	0.311 *	−0.203 *ms*	0.205 *ms*			0.313 *
AI dependency→Intrinsic motivation						
External regulation→Identified regulation						
External regulation→Intrinsic motivation	0.301 *					
Identified regulation→Intrinsic motivation	−0.454 **				−0.473 ***	−0.549 ***

Note: *ms* = *p* < 0.10, * *p* < 0.05, ** *p* < 0.01, *** *p* < 0.001. Since external regulation only passed the invariance test between T1–T2 and T2–T4, the path from External regulation to Intrinsic motivation cannot be compared for group differences between T3–T4 and T1–T4.

**Table 8:** Holm-Adjusted Pairwise Cross-Wave Comparisons of Direct and Indirect Proportions.

Comparison	Proportion Type	Mean Difference	95% CI	Raw *p*	Holm *p*	Conclusion
T2 vs. T1	Direct	−0.502	[−0.932, −0.102]	0.018	0.18	n.s.
	Indirect	+0.502	[0.102, 0.932]	0.018	0.18	n.s.
T3 vs. T1	Direct	−0.397	[−0.813, 0.012]	0.055	0.434	n.s.
	Indirect	+0.397	[−0.012, 0.813]	0.055	0.434	n.s.
T4 vs. T1	Direct	−0.903	[−1.664, −0.379]	0.001	0.012	sig.
	Indirect	+0.903	[0.379, 1.664]	0.001	0.012	sig.
T3 vs. T2	Direct	−0.105	[−0.282, 0.511]	0.588	1	n.s.
	Indirect	+0.105	[−0.511, 0.282]	0.588	1	n.s.
T4 vs. T2	Direct	−0.401	[−1.127, 0.117]	0.127	0.507	n.s.
	Indirect	+0.401	[−0.117, 1.127]	0.127	0.507	n.s.
T4 vs. T3	Direct	−0.505	[−1.248, 0.007]	0.054	0.434	n.s.
	Indirect	+0.505	[−0.007, 1.248]	0.054	0.434	n.s.

Note: Holm-adjusted *p*-values are presented for pairwise cross-wave comparisons of direct- and indirect-effect proportions. sig = significant (*p* < 0.05), n.s. = not significant (*p* ≥ 0.05).

**Table 9:** Bootstrap Difference Test of Direct and Indirect Effect Proportions for T1 and T4.

Time Point	Direct Proportion [95% CI]	Indirect Proportion [95% CI]
T1	84.6% [51.3%, 112.8%]	15.4% [−12.8%, 48.7%]
T4	−5.7% [−75.8%, 32.8%]	105.7% [67.2%, 175.8%]
T4 vs. T1 difference	−90.3% [−166.4%, −37.9%], *p* = 0.001	+90.3% [37.9%, 166.4%], *p* = 0.001

**Table 10:** Comparison of path coefficients (T1–T4).

Path	T1 Model	T2 Model	T3 Model	T4 Model
AI dependency→AI-assisted creativity	0.180 ns	0.057 ns	0.168 **	−0.031 ns
AI dependency→External regulation	0.479 ***	0.445 ***	0.585 ***	0.560 ***
AI dependency→Identified regulation	0.177 **	−0.134 ns	0.069 ns	−0.136 ns
AI dependency→Intrinsic motivation	−0.113 ns	−0.018 ns	−0.066 ns	−0.043 ns
External regulation→Identified regulation	0.584 ***	0.488 ***	0.483 ***	0.496 ***
External regulation→Intrinsic motivation	0.465 ***	0.164 *	0.253 **	0.001 ns
Identified regulation→Intrinsic motivation	0.139 ns	0.592 ***	0.612 ***	0.687 ***
External regulation→AI-assisted creativity	0.033 ns	0.148 *	0.022 ns	0.135 *
Identified regulation→AI-assisted creativity	0.314 *	0.451 ***	0.380 ***	0.420 ***
Intrinsic motivation→AI-assisted creativity	0.394 ***	0.310 ***	0.388 ***	0.373 ***

Note: *** *p* < 0.001, ** *p* < 0.01, * *p* < 0.05, ns = not significant (*p* ≥ 0.05).

**Table 11:** Mediating path coefficient analysis (T1–T4).

Path	T1 Model	T2 Model	T3 Model	T4 Model
AI dependency→External regulation→AI-assisted creativity	0.016 ns	0.066 ns	0.013 ns	0.076 *
AI dependency→dentified regulation→AI-assisted creativity	0.056 ns	−0.060 ns	0.026 ns	−0.057 ns
AI dependency→Intrinsic motivation→AI-assisted creativity	−0.045 ns	−0.006 ns	−0.026 ns	−0.016 ns
AI dependency→External regulation→dentified regulation→AI-assisted creativity	0.088 *	0.098 ***	0.107 ***	0.117 ***
AI dependency→External regulation→Intrinsic motivation→AI-assisted creativity	0.088 *	0.023 ns	0.057 *	0.000 ns
AI dependency→dentified regulation→Intrinsic motivation→AI-assisted Creativity	0.010 ns	−0.025 ns	0.016 ns	−0.035 ns
AI dependency→External regulation→dentified regulation→Intrinsic motivation→AI-assisted creativity	0.015 ns	0.040 **	0.067 **	0.071 ***

Note: *** *p* < 0.001, ** *p* < 0.01, * *p* < 0.05, ns = not significant (*p* ≥ 0.05).

**Table 12:** Wave-wise comparisons of composite means for each scale (observed-level).

Variables	T1 (*n* = 124)	T2 (*n* = 180)	T3 (*n* = 258)	T4 (*n* = 226)
AI-assisted creativity	3.990	3.599	3.543	3.440
AI dependency	3.302	3.034	3.036	3.070
External regulation	3.522	3.265	3.182	3.238
Identified regulation	4.013	3.820	3.800	3.618
Intrinsic motivation	3.605	3.565	3.479	3.450

**Table 13:** Differences relative to T1, significance, and effect sizes (ANCOVA adjusting for gender, year of study, and major).

Variables	Comparisons	Differences	ANCOVA b	SE	Holm *p*	d
AI-assisted creativity	T2–T1	−0.391	−0.368	0.079	<0.001	−0.61
	T3–T1	−0.446	−0.427	0.079	<0.001	−0.67
	T4–T1	−0.549	−0.516	0.075	<0.001	−0.95
AI dependency	T2–T1	−0.267	−0.277	0.103	0.059	−0.30
	T3–T1	−0.266	−0.286	0.103	0.054	−0.31
	T4–T1	−0.232	−0.251	0.098	0.064	−0.31
External regulation	T2–T1	−0.257	−0.242	0.090	0.059	−0.37
	T3–T1	−0.340	−0.365	0.090	0.001	−0.45
	T4–T1	−0.284	−0.240	0.086	0.054	−0.47
Identified regulation	T2–T1	−0.193	−0.128	0.090	0.550	−0.27
	T3–T1	−0.214	−0.186	0.090	0.199	−0.30
	T4–T1	−0.395	−0.313	0.086	0.003	−0.62
Intrinsic motivation	T2–T1	−0.040	−0.013	0.100	0.899	−0.05
	T3–T1	−0.126	−0.148	0.100	0.550	−0.14
	T4–T1	−0.155	−0.116	0.095	0.550	−0.20

Note: Differences represent composite-mean deviations of later-wave samples relative to the T1 baseline sample; b denotes ANCOVA estimates after adjusting for gender (1 = male, 2 = female), year of study (1 = first-year to 5 = postgraduate, reference = first-year), and major (1 = humanities, 0 = science); Holm correction was applied across all 15 comparisons; d is Cohen’s d without covariate adjustment.

**Table 14:** Qualitative evidence supporting mechanisms underlying key theoretical propositions.

Key Proposition	Theme from Interviews	Illustrative Evidence	Salience/Context
Associations among different types of motivation	Widespread engagement triggered by the broad diffusion of generative AI facilitated intensive usage, enabled value discovery, and brought about a pragmatic realignment of motivations.	S2: “(At T1) it was purely out of curiosity; I wanted to see what kind of answers it could give… (After T2) it became a habit, because its functions in summarizing and providing support were really convenient.” P4: “Everyone was talking about it when it went viral, so I tried it (external event)… Later I found it could help me a lot with my studies, such as outlining course papers and explaining complex concepts, and I gradually became reliant on it.” S11: “(At T1) I was just following the trend… but (starting at T2) I found it worked better than typical homework aids and could respond to open-ended questions. So it shifted from ‘just for fun’ to ‘regular use’.”	The vast majority of respondents experienced an initial extrinsic trigger, followed by the development of value acceptance. The peak of technological diffusion constituted a critical trial window, during which users rapidly recognized the instrumental value of AI for themselves through hands-on use.
Differentiation among different types of motivation	Hierarchy of usage motivations: extrinsic motivations such as following trends, seeking convenience, and meeting basic task requirements represent a superficial starting point, whereas the drivers sustaining continued use include problem-solving, cognitive growth, and interest-driven exploration.	S3: “Initially, I adopted AI simply because others were using it… Over time, however, I came to rely on it primarily because it was more efficient than Baidu and provided direct responses, which reflects identified regulation.” P7: At first, I was driven purely by curiosity, representing intrinsic motivation. However, after recognizing its capacity to assist with data processing and code generation while significantly improving efficiency, I developed sustained reliance. This transition illustrates identified regulation. S8: “At the outset, I was drawn to it for entertainment such as fortune-telling, driven by intrinsic motivation. After T3, I confined its use to recreational purposes including browsing recipes and creating GIFs, and no longer regard it as reliable for academic work.”	Extrinsic drivers predominated in the initial phase (following trends, task-related pressure). Upon entering the mature phase, students who relied on extrinsic drivers tended to discontinue or reduce their AI use, whereas sustained users placed increasing emphasis on AI’s practical value (identified regulation) or exploratory enjoyment (intrinsic motivation).
Associations between AI dependency and creativity	Cognitive Shift: In the early stage, users experienced cognitive outsourcing and cognitive empowerment; in the middle and later stages, they expressed concerns about the decline in their own in-depth thinking and innovative abilities.	S1: “I learned logical thinking from AI, which made my thinking more comprehensive and structured… But later I wondered whether my own mind would become less capable if I kept using it.” P1: “I think it limits my thinking… After using it, I find it hard to come up with extra ideas of my own.” S5: “At first I thought it was really smart… But later I felt it actually boxes in my thinking, turning my originally active and open way of thinking into something narrow and restricted.”	Among the 20 university students, more than 80% explicitly voiced concerns that AI dependency may lead to cognitive inertia and reduced creative flexibility. This shift often coincides with disappointment toward AI and subsequent reflective processes.
Mapping to the Gartner Hype Cycle: External factors drive the trajectory of the curve, while internal practical value drives sustained usage.	Roller-coaster-like experiences and mental maturation: emotional experiences toward AI shift from high initial expectations to subsequent disappointment, yet users remain reluctant to discontinue use—what emerges is a pragmatic rebound following rational evaluation.	S9: “At T2, I thought it was extremely intelligent, almost like a real person…At T3, I was thoroughly disappointed when I discovered it fabricated academic references, shattering my positive impression. At T4, I have gained a clear understanding of its strengths and limitations. I therefore only use it for tasks it performs well and that meet my actual needs.” P8: “When it went viral at T2, I thought it was omnipotent. Later at T3, I grew cautious after realizing it often made up information… Now at T4, I regard it as an advanced search engine and translator, with a calm and rational mindset.” S7: “Speaking of the curve, I think it is highly consistent with my experience… No matter how people hype or criticize it, I will continue using it as long as it practically helps me solve my academic tasks.”	Nearly all respondents’ narratives revealed high initial expectations, followed by more rational adjustments upon discovering AI’s limitations. The stabilized usage pattern was no longer shaped by hype-driven enthusiasm, but by validated, personalized judgments of practical value.
Evolution of Mean Values: Dependency and intrinsic motivation declined, while external regulation remained stable. This is a counterintuitive and unexpected finding.	As the hype subsides and reliance diminishes, the instrumental rationality of convenience remains.	S4: “When it was popular at T2, I asked it about everything and relied heavily on it. Later at T3, I used it less after finding it made up information. However, my intention to use it for convenience and task completion has never changed.” P5: “The novelty wore off quickly, showing a decline in intrinsic motivation. But for formatted tasks requiring quick results such as making templates, I still turn to it first, indicating stable external regulation.”	Alongside the fading of novelty, statements such as “I still use it when necessary” and “after all, it saves me time” attest to the persistence of external regulation.

## Data Availability

The data presented in this study are available from the corresponding author upon reasonable request and are also available at the Open Science Framework (OSF) at https://osf.io/t4hzw/overview?view_only=4ed50a397c394c5cae7c4d3254a4f8aa, accessed on 1 November 2025.

## Appendix A

### Appendix A.1. Six Sensitivity-Check Strategy Analyses

**Analyses**	***n***	**b**	**Holm *p***
S1 Full-sample ANCOVA	788	−0.516	2.0 × 10^−10^
S2a Humanities-only subsample	603	−0.551	2.9 × 10^−10^
S2b Female-only subsample	640	−0.478	4.5 × 10^−8^
S2c First- & second-year undergraduates-only subsample	448	−0.584	4.0 × 10^−10^
S2d Humanities-female-only subsample	517	−0.499	7.1 × 10^−8^
S3 Propensity-score weighting	788	−0.650	1.5 × 10^−27^

### Appendix A.2. Robustness Assessment

**Variables**	**Robustness Across the Six Specifications**	**Robustness** **Classification**
AI-assisted creativity	8/18 significant; b = −0.478 to −0.650	Highly robust
Identified regulation (T4)	6/6 significant; (p_holm = 0.00098 to 0.021)	Robust
External regulation (T3)	6/6 significant; (p_holm = 0.00066 to 0.013)	Robust
AI dependency	Only 2/6 significant (S2c, S3); remaining four p_holm = 0.064~0.40	Non-robust
Intrinsic motivation	0/6 significant	No meaningful shift

## Appendix B. Cross-Time Multi-Group Analysis Results

**Panel A: MICOM Step1—Configural invariance**												
Construct	T1			T2			T3			T4		
AI dependency	Established			Established			Established			Established		
AI-assisted creativity	Established			Established			Established			Established		
External regulation	Established			Established			Established			Established		
Identified regulation	Established			Established			Established			Established		
Intrinsic motivation	Established			Established			Established			Established		
**Panel B: MICOM Step2—compositional invariance**												
Construct	T1–T2		T1–T3		T1–T4		T2–T3		T2–T4		T3–T4	
	c	*p*	c	*p*	c	*p*	c	*p*	c	*p*	c	*p*
AI dependency	1.000	0.949	0.999	0.368	0.999	0.795	1.000	0.972	0.998	0.538	0.999	0.368
AI-assisted creativity	1.000	0.998	1.000	0.340	0.999	0.384	0.999	0.400	0.999	0.317	1.000	0.340
External regulation	0.986	0.134	0.993	0.000	0.974	0.014	0.997	0.023	0.990	0.066	0.993	0.000
Identified regulation	1.000	0.847	1.000	0.402	1.000	0.563	1.000	0.609	1.000	0.898	1.000	0.402
Intrinsic motivation	1.000	0.727	0.999	0.128	1.000	0.709	1.000	0.715	0.998	0.176	0.999	0.128
**Panel C: MICOM step 3a—equality of composite mean values**												
Construct	T1–T2		T1–T3		T1–T4		T2–T3		T2–T4		T3–T4	
	Diff	*p*	Diff	*p*	Diff	*p*	Diff	*p*	Diff	*p*	Diff	*p*
AI dependency	0.300	0.012	−0.053	0.548	0.311	0.004	0.002	0.974	−0.051	0.627	−0.053	0.548
AI-assisted creativity	0.611	0.000	0.185	0.050	0.904	0.000	0.085	0.397	0.296	0.001	0.185	0.050
External regulation	0.407	0.000	−0.083	0.351	0.569	0.000	0.114	0.230	0.044	0.672	−0.083	0.351
Identified regulation	0.265	0.023	0.263	0.011	0.599	0.000	0.028	0.756	0.291	0.004	0.263	0.001
Intrinsic motivation	0.044	0.693	0.053	0.575	0.222	0.052	0.120	0.230	0.195	0.044	0.053	0.575
**Panel D: MICOM step3b—equality of composite variances**												
Construct	T1–T2		T1–T3		T1–T4		T2–T3		T2–T4		T3–T4	
	Diff	*p*	Diff	*p*	Diff	*p*	Diff	*p*	Diff	*p*	Diff	*p*
AI dependency	0.114	0.470	0.544	0.001	0.780	0.000	0.118	0.396	0.657	0.000	0.544	0.001
AI-assisted creativity	0.224	0.222	0.535	0.016	0.564	0.000	−0.181	0.444	0.351	0.027	0.535	0.016
External regulation	−0.146	0.393	0.669	0.000	0.230	0.214	−0.211	0.190	0.444	0.007	0.669	0.000
Identified regulation	−0.291	0.124	0.324	0.093	0.102	0.450	0.074	0.727	0.399	0.007	0.324	0.093
Intrinsic motivation	0.702	0.001	0.500	0.003	1.048	0.000	−0.141	0.442	0.359	0.018	0.500	0.003
